# Immune restoration in HIV-1-infected patients after 12 years of antiretroviral therapy: a real-world observational study

**DOI:** 10.1080/22221751.2020.1840928

**Published:** 2020-12-07

**Authors:** Jiaye Liu, Lifeng Wang, Yuying Hou, Yan Zhao, Zhihui Dou, Ye Ma, Dawei Zhang, Yasong Wu, Decai Zhao, Zhongfu Liu, Fujie Zhang, Lei Jin, Ji-Yuan Zhang, Ruonan Xu, Ming Shi, Lei Huang, Zunyou Wu, Mengjie Han, George F. Gao, Fu-Sheng Wang

**Affiliations:** a National Center for AIDS/STD Control and Prevention, China Center for Disease Control and Prevention, Beijing, People’s Republic of China; b Treatment and Research Center for Infectious Diseases, The Fifth Medical Center of PLA General Hospital, Beijing, People’s Republic of China; c Department of liver disease, Second Hospital Affiliated to Southern University of Science and Technology, Shenzhen Third People’s Hospital, Shenzhen, People’s Republic of China

**Keywords:** HIV, CD4, CD8, CD4/CD8 ratio, immune restoration

## Abstract

Using normalization of CD4 counts as the main evaluation parameter of complete immune restoration for HIV-1 patients under antiretroviral therapy (ART) might be not enough. A comprehensive evaluation system more accurately reflecting immune restoration are urgently needed. Totally, 91,805 HIV-1 patients from 17 tertiary hospitals in China during 2005–2018 were included in this study. Immune restoration and mortality were assessed. Patients initiated ART with baseline CD4 counts <50, 50–199, 200–349, 350–499, and ≥500 cells/μL, and results showed an increase in the median CD4 counts to 445 (12-year), 467 (12-year), 581 (11-year), 644 (7-year), and 768 cells/µL (5-year), as well as the CD4/CD8 ratio to 0.59 (12-year), 0.65 (12-year), 0.79 (11-year), 0.82 (7-year), 0.9 (5-year), respectively. The median CD8 count was relatively high (median range 732–845 cells/μL), regardless of the baseline CD4 counts. Furthermore, the probabilities of death in patients achieving CD4 counts ≥500 cells/μL and CD4/CD8 ratio ≥0.8 simultaneously were significantly lower than those in patients achieving either CD4 counts ≥500 cells/μL (2.77% vs 3.50%, *p*=0.02) or CD4/CD8 ≥ 0.8 (2.77% vs 4.28%, *p*<0.001) after 12-year of ART. In this study, a new binary-indicator would accurately assess immune restoration in the era of “treat all.”

## Introduction

It is well known that HIV-1 infection mainly attacks CD4 cells and induces progressive loss of CD4 counts [1]. Alteration in CD4 counts during the treatment of HIV-1 infection is significantly associated with immune restoration, morbidity, and mortality [2–4]. Although patients are more likely to achieve normalized CD4 counts through early initiation of ART, overactivation and expansion of CD8 T cells, as well as systemic inflammation are observed in such patients. Furthermore, a few studies have reported that there is an U-shaped relationship between CD8 counts and all-cause mortality [5], and a low CD4/CD8 ratio is associated with increased AIDS- or non-AIDS-related morbidity and mortality in patients on ART after adjusting the CD4 count [6,7]. The CD4 counts, CD8 counts, and CD4/CD8 ratio are considered to reflect immune status and predict different outcomes. In the era of “treat all”, restoration of CD4 cell counts (≥500 cells/μL) alone, which was previously accepted as a satisfactory treatment outcome indicator, may not be sufficient for clinical practices. Hence, it is necessary to establish a comprehensive immune assessment system integrating additional immune parameters.

Based on data from previous large-scale prospective studies and recommendations from the World Health Organization (WHO), there are four CD4 cut-off values to start ART for people living with HIV-1 (PLWH) in China [8]. Since 2002, PLWHs with CD4 counts <200 cells/μL have been eligible for free ART. From 2008 to 2013, this criterion was modified to <350 cells/μL, and then to <500 cells/μL until 2015. Since 2016, all PLWHs are eligible for free ART, regardless of the CD4 count. So far, based on China's National Free Antiretroviral Treatment Program (NFATP) database, a series of analyses have been performed to explore the efficacy of ART in adult PLWH in China [9–12]. These studies indicated that the scale-up of ART and earlier initiation of therapy had significantly reduced the mortality rate among those patients. However, they mainly analyzed participants who received free ART before 2012 and had low CD4 counts at ART initiation, mortality as the main outcome, and an evaluation period of under 5 years. Hence, a comprehensive evaluation of immune restoration and assessment of >10-year disease outcomes in national patients who received free ART in China are still lacking.

To address the above-mentioned issues, this real-world observational study aimed to: (1) analyze the dynamics of CD4 counts, CD8 counts, and CD4/CD8 ratio during long-term ART, (2) investigate the cumulative probability of immune restoration and mortality and identify their associated factors, and (3) explore a comprehensive evaluation system for immune restoration.

## Methods

### Study design and patients

**Figure 1.**
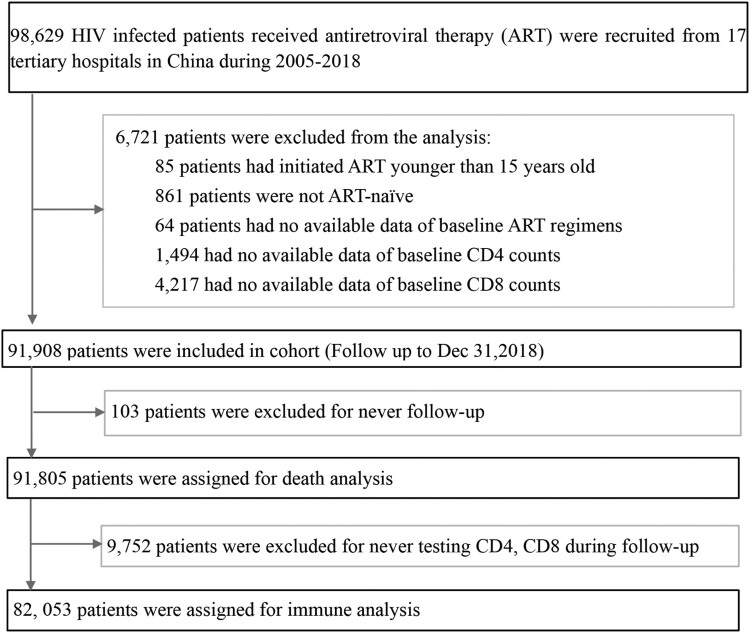
Enrollment of HIV-1-infected patients initiated on ART between Jan 1, 2005 and Jun 30, 2018.

This real-world cohort study was based on existing records in the NFATP database, including data on HIV-1-infected patients who received free ART with long-term follow-up care in China [13,14]. We included the patients if they were older than 15 years of age, treatment-naive, and started ART between Jan 1, 2005 and Jun 30, 2018. The locations of the 17 hospitals are presented in Appendix 1. The following patients were excluded from this study: (1) patients from Taiwan province, Hong Kong and Macau Special Administration Regions, and other foreign countries, (2) patients without ART initiation regimen, or baseline CD4 counts, or baseline CD8 counts, and (3) patients without any follow-up records. Finally, we established two cohort sets: death analysis set and immune analysis set (Figure 1). Baseline information and follow-up records, including demographic, clinical characteristics, and laboratory test data were collected from local healthcare workers using standardized case report forms. This study was approved by our Research Ethics Committee, and written informed consent was obtained from each patient.

### Procedure

We assessed two main outcomes: immune restoration and death. Follow-up data were included up to Dec 31, 2018. Patients were considered lost to follow-up if they missed follow-up ≥90 days. For each participant, the longest observation duration was 12 years from the date of ART initiation.

Immune restoration was indicated by CD4 counts ≥200, ≥350, and ≥500 cells/μL, CD4/CD8 ratio ≥0.4 and ≥0.8, or simultaneous achievement of CD4 counts ≥500 cells/µL and CD4/CD8 ratio ≥0.8 among patients with different baseline CD4 counts. The observation period was from the date of ART initiation to the date of occurrence of outcomes. Patients were censored at death, loss to follow-up, transfer to other facilities, or the last follow-up visit before Dec 31, 2018.

We also assessed the survival rate by measuring the time from the initiation of ART to death (all-cause) or censoring. Patients were censored at loss to follow-up, transfer to other facilities, or the last follow-up visit.

### Statistical analysis

We conducted three analyses. First, we assessed the dynamics of CD4 counts, CD8 counts, CD4/CD8 ratio at baseline and different time points after ART initiation stratified by the baseline CD4 counts: <50, 50–199, 200–349, 350–499, and ≥500 cells/μL. Second, we assessed the probability of CD4 cell counts recovery, CD4/CD8 ratio restoration, and binary-indicator immune restoration (CD4 counts ≥500 cells/µL and CD4/CD8 ratio ≥0.8), and explored the factors associated with achieving these events. We estimated these probabilities using Kaplan-Meier analysis stratified by the baseline CD4 counts, and determined statistical significance using the log-rank test for running parallel hazard rates and a two-stage procedure for crossing hazard rates [15]. Third, we assessed the probability of death using Kaplan-Meier analysis and explored factors associated with death using the Cox proportional model. The detail of three analyses was described in Appendix 2.

All significance tests performed were two-sided. *P* values less than 0.05 were deemed statistically significant and 95% confidence interval (CI) was calculated for point estimates. All analyses were carried out using SAS software version 9.4 (SAS Institute) or SPSS version 23.0.

## Results

### Baseline characteristics of PLWH

Baseline characteristics of two analyses sets among the 91,805 enrolled cases are summarized in Table 1. In mortality analysis, the mean age of PLWH was 36.91 years and 74,719 (81.39%) patients were male. In immune analysis, the mean age of PLWH was 36.49 years and 66,889 (81.52%) patients were male. The median baseline CD4 counts, CD8 counts, and CD4/CD8 ratio were similar between immune and mortality analysis. Baseline CD4 counts <200 cells/µL were observed in 45.49% of patients in mortality analysis and 44.21% in immune analysis (Table 1). In China, plasma HIV RNA monitoring is free for each patient at least once per year. In the NFATP database, there were 64,083 patients on ART who had better viral load records during the follow-up period, and 61,379 of them (95.78%) were found to achieve full viral suppression (HIV-1 RNA <400 copies/mL) [16].

**Table 1.** Baseline features of HIV-1-infected patients.

Characteristics	Death Analysis Set	Immune Analysis Set
Overall, *n*	91805	82,053
Sex, male	74,719 (81.39)	66,889 (81.52)
Age at Start ART (year), mean ± SD	36.91 ± 12.61	36.49 ± 12.28
Route of HIV infection		
Injection drug use	5771 (6.29)	4664 (5.68)
MSM	40,511 (44.13)	37,438 (45.63)
Heterosexual	40,366 (43.97)	35,405 (43.15)
Blood transfusion	529 (0.59)	481 (0.59)
Other/Unknown	4628 (5.04)	4065 (4.95)
WHO clinical stage, III–IV	41,373(45.07)	35,970 (43.84)
Baseline Hb (mg/dL), <90	5938 (6.47)	4618 (5.63)
Baseline CD4 (cells/μL)		
Median (IQR)	221(79-339)	226 (88-341)
<50	18,069 (19.68)	15,158 (18.47)
50–199	23,698 (25.81)	21,119 (25.74)
200–349	28,794 (31.36)	26,481 (32.27)
350–499	14,141 (15.40)	12,937 (15.77)
≥500	7103 (7.74)	6358 (7.75)
Baseline CD8 (cells/μL)		
Median (IQR)	819 (531-1188)	832 (545-1200)
<500	20,623 (22.46)	17,493 (21.32)
500∼999	37,981 (41.37)	34,201 (41.68)
≥1000	33,201 (36.16)	30,359 (37.00)
Baseline CD4/CD8 ratio		
Median (IQR)	0.23 (0.11-0.37)	0.24 (0.12-0.37)
<0.1	20,330 (22.14)	17,323 (21.11)
0.10–0.19	19,194 (20.91)	17,262 (21.04)
0.20–0.39	32,828 (35.76)	29,984 (36.54)
0.40–0.79	17,184 (18.72)	15,511 (18.90)
≥0.80	2269 (2.47)	1973 (2.40)
Initial antiretroviral regimen		
AZT+3TC + NVP/EFV	18,196 (19.82)	16,787 (20.46)
D4T+3TC + NVP/EFV	8949 (9.75)	7852 (9.57)
TDF+3TC + NVP/EFV	53,740 (58.54)	48,401 (58.99)
TDF+3TC + LPVr	2696 (2.94)	2215 (2.70)
Other	8224 (8.96)	6798 (8.28)
Calendar year at baseline		
2005–2007	3164 (3.45)	2609 (3.18)
2008–2013	28,886 (31.46)	26,447 (32.23)
2014–2015	25,056 (27.29)	23,347 (28.45)
2016–2018	34,699 (37.80)	29,650 (36.14)

Notes: Data are expressed as n (%) or median (IQR). ART: antiretroviral therapy; SD: standard deviation; IQR: interquartile range; HIV: human immunodeficiency virus; MSM: men who have sex with men; Hb: hemoglobin; TDF: tenofovir disoproxil fumarate; 3TC: lamivudine; D4 T: stavudine; EFV: efavirenz; NVP: nevirapine; AZT: zidovudine; LPVr: lopinavir/ritonavir.

### The dynamics of CD4 counts, CD8 counts, CD4/CD8 ratio in PLWH on ART

**Figure 2.**
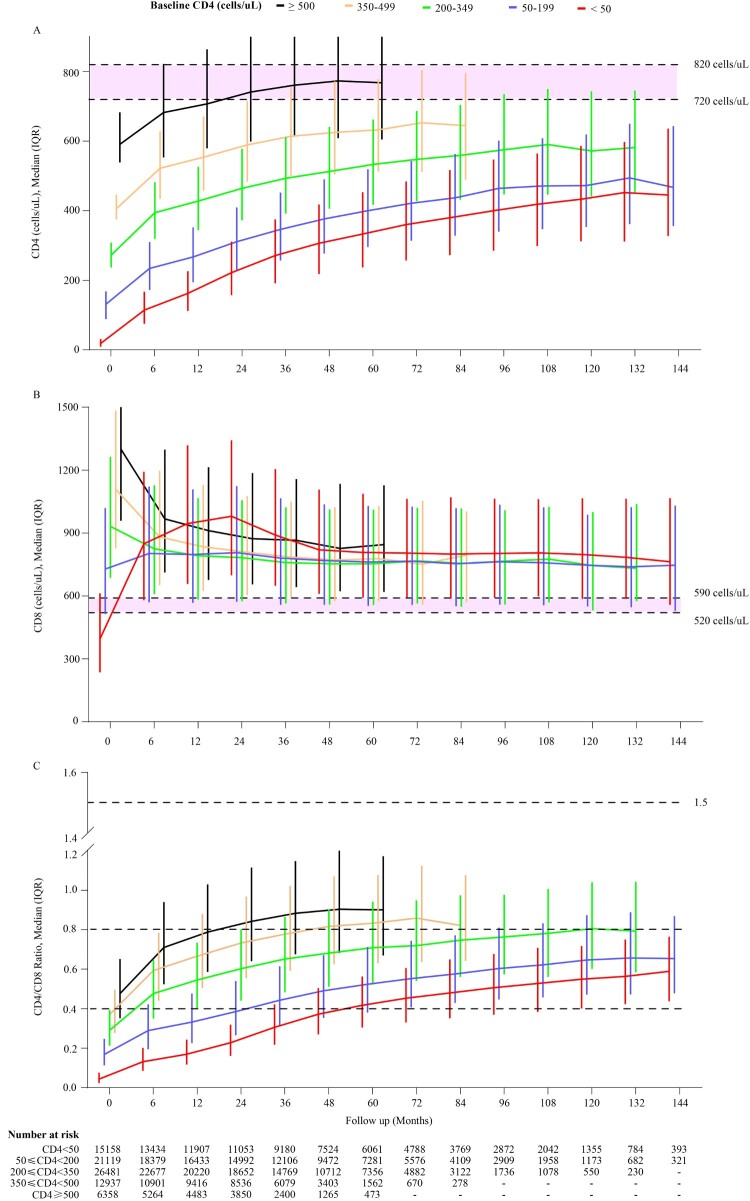
The dynamic trajectories of CD4 counts, CD8 counts, CD4/CD8 ratio in HIV-1 patients based on five groups of baseline CD4 counts over a 12 years follow-up period. Although the CD4 counts were quickly restored to above 500 cells/µL after ART in patients with baseline CD4 counts ≥350 cells/µL, in advanced HIV-1 patients (CD4 counts <200 or <50 cells/μL), the restoration of CD4 counts was slower and more difficult to reach 500 cells/μL even after 12 years of ART (A). HIV-1-infected patients on ART usually displayed high levels of CD8 counts (B), and rarely showed restoration of normal CD4/CD8 ratio(C) during the 12-year follow-up among patients with different baseline CD4 counts. The pink shaded areas in A and B represent a range of mean CD4 and CD8 counts in Chinese healthy population from different researches, respectively. Dash symbol in “Number at risk” means that when the numbers of patients were less than 200 in the corresponding follow-up points, we will not exhibit the trajectories. IQR = inter quartile range.

**Figure 3.**
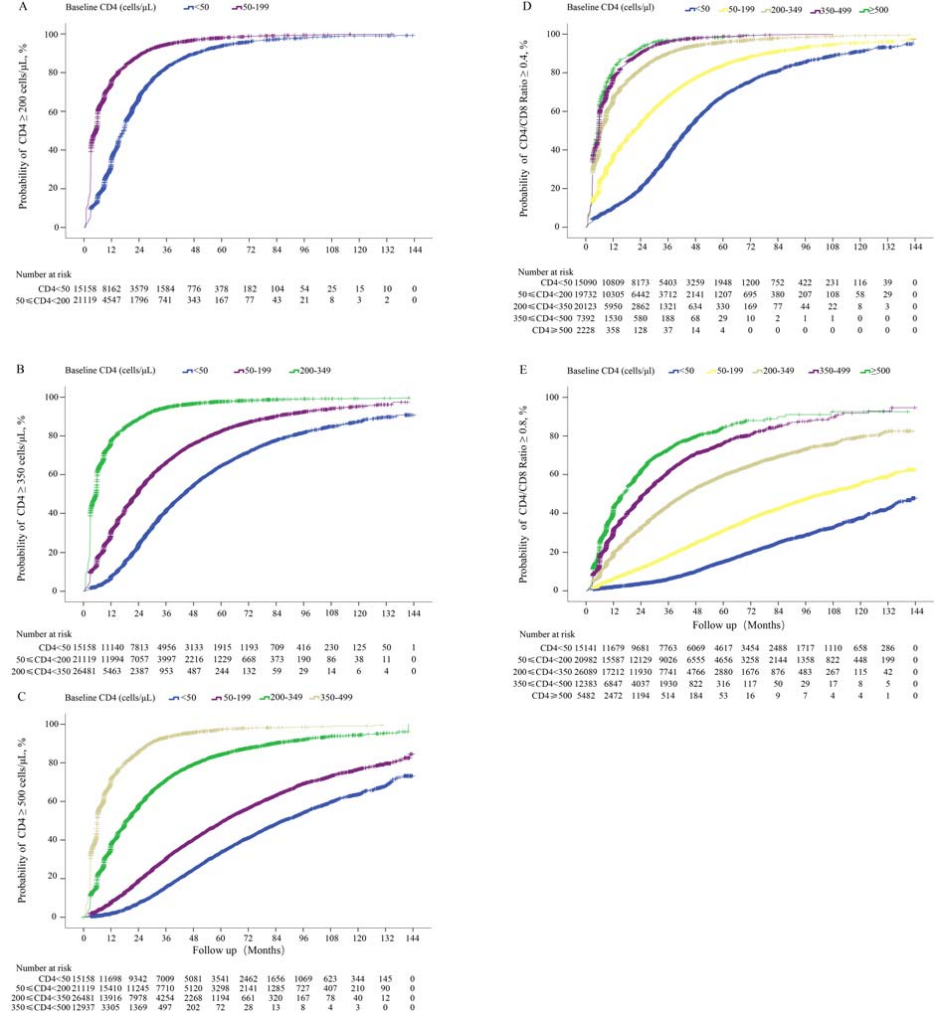
The cumulative probability of restoration of CD4 count and CD4/CD8 ratio in HIV-1-infected patients after ART initiation. Analyses of the cumulative probability of CD4 counts reaching above 200 (A), 350 (B), and 500 cells/μL (C). The cumulative probability of CD4/CD8 ratio reaching above 0.4 (D) and 0.8 (E).

The mean CD4 counts in healthy Chinese individuals ranges between 720–820 cells/µL [17–19]. Here, ART was shown to effectively restore CD4 counts in PLWH with different baseline CD4 counts, especially within the first 6 months of ART initiation (Figure 2(A) and Figure 3(A–C)). After 5 years of ART, the median CD4 counts reached 333 (237–453), 400 (296–519), 533 (417–662), 632 (513–778), and 768 cells/µL (605–947) in the five groups with baseline CD4 counts <50, 50–199, 200–349, 350–499, and ≥500 cells/µL, respectively. The corresponding values persistently increased to 433 (312–586), 472 (353–619), and 572 cells/µL (441–743) in patients with baseline CD4 counts <50, 50–199, and 200–349 cells/µL, respectively, at 10 years after ART initiation. However, after a 12-year follow-up period, the median CD4 count did not reach 500 cells/µL in patients with baseline CD4 counts <200 cells/µL.

Healthy Chinese subjects usually have mean CD8 counts of 520–590 cells/µL [17–19]. In this study, in patients with baseline CD4 counts <50 cells/µL, the median CD8 count rapidly increased to 849 cells/µL (580–1192) within the first 6 months of ART initiation, gradually reaching a peak of 980 cells/µL (698–1342) after a 2-year follow-up period, and then slowly decreased and stabilized at around 800 cells/µL. However, in patients with baseline CD4 counts of 50–199 cells/µL, the CD8 counts slightly increased from 720 to 810 cells/µL after ART and then maintained steady levels. All ART patients with baseline CD4 counts of 200–349, 350–499, and ≥500 cells/µL showed a significantly decreasing trajectory within the first year of ART, and then stabilized at around 750–850 cells/µL at 2 or 3 years after ART. In general, CD8 counts remained higher in all patients than in healthy controls regardless of the baseline CD4 counts (Figure 2(B)).

The mean CD4/CD8 ratio among healthy people has been reported to be around 1.5 [17–19]. In this study, we found that the CD4/CD8 ratio rapidly and significantly increased to 0.48, 0.59, and 0.71 within the first 6 months of ART and reached 0.71, 0.83, and 0.90 after 5 years of ART in the patients with baseline CD4 counts of 200–349, 350–499, and ≥500 cells/µL, respectively. While the ratio gradually increased to 0.13 and 0.29 within the first 6 months of ART and reached 0.59 and 0.65 after 12 years of ART in the patients with baseline CD4 counts <50 and 50–199 cells/µL, respectively. In general, not all ART patients achieved a CD4/CD8 ratio ≥1.0 even at the longest follow-up duration of 12 years (Figure 2(C)).

### The cumulative probability of immune restoration and its associated factors in HIV-1 patients on ART

In this study, we found that 90% of all subjects with baseline CD4 counts <50 cells/µL achieved CD4 counts ≥200 cells/µL at 6 years after ART (Figure 3(A)). Around 90% of all subjects with baseline CD4 counts <50 and 50–199 cells/µL achieved CD4 counts ≥350 cells/µL at the end of 12 years of ART (Figure 3(B)). While subjects with baseline CD4 counts <50, 50–199, 200–349, and 350–499 cells/μL achieved CD4 counts ≥500 cells/μL at probabilities of 1.97, 7.84, 62.85, and 71.07%, respectively, at 12 months after ART. The corresponding cumulative probabilities were 63.68, 76.64, and 94.5% in patients with baseline CD4 counts <50, 50–199, 200–349 cells/µL, respectively, after 10 years of ART. At the longest 12-year follow-up duration, only 73.19 and 84.48% of the surviving subjects with baseline CD4 counts <50 and 50–199 cells/µL, respectively, achieved CD4 counts ≥500 cells/µL. Patients with baseline CD4 counts ≥350 cells/µL could more easily reach CD4 counts ≥500 cells/μL within 6 months of ART (Figure 3(C)). In addition, we also calculated the time taken by 50 or 80% (probability) of patients with different baseline CD4 counts to achieve different CD4 recovery counts (e.g. ≥200, ≥350, and ≥500 cells/uL), which provides useful data for physicians (Appendix 3). Considering the restoration of CD4/CD8 ratio, we found that the higher the baseline CD4 counts, the easier it was to achieve CD4/CD8 ratios ≥0.4 (Figure 3(D)) and ≥0.8 (Figure 3(E)).

In the multivariate model, females, younger age, men who have sex with men (MSM), higher baseline CD4 counts and CD8 counts, hepatitis B surface antigen (HBsAg) negative, and protease inhibitor (PI)-based regimens were strongly associated with CD4 counts ≥500 cells/μL (Appendix 4). While lower CD8 counts were associated with CD4/CD8 ratios ≥0.4 and ≥0.8 (Appendix 5, 6).

### The cumulative probabilities of death and its associated factors in HIV-1-Infected patients on ART

**Figure 4.**
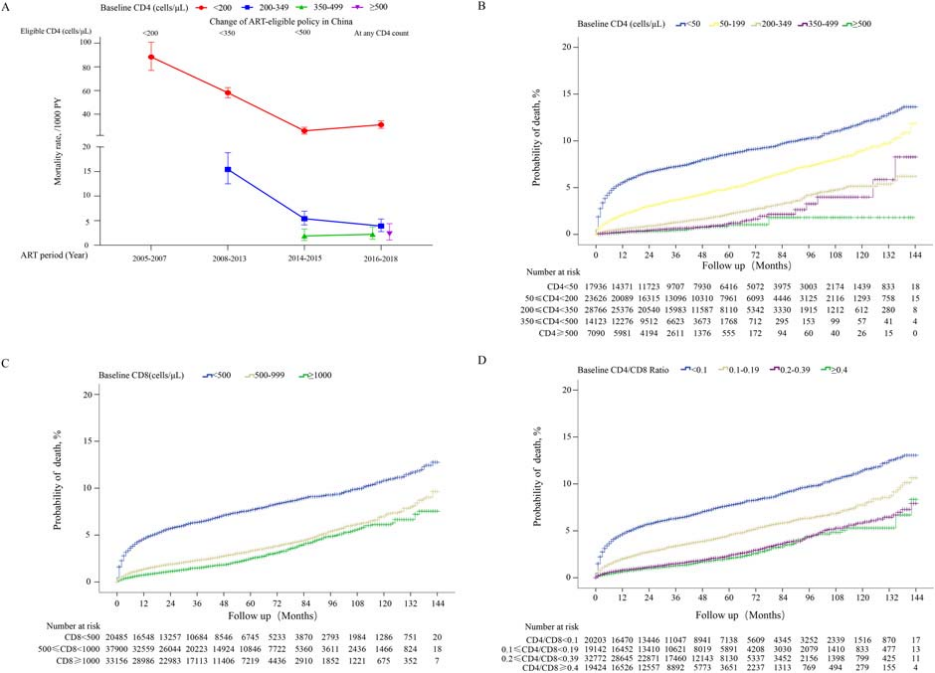
Cumulative probability of death among HIV-1-infected patients on ART. Estimation of changes in mortality rates in patients with different baseline CD4 counts during the first year of ART based on different ART eligibility criteria (A). Cumulative probability of death stratified by different baseline CD4 counts (B), different baseline CD8 counts (C), and different baseline CD4/CD8 ratios (D).

In this dynamic cohort, 3,134 deaths from 91,805 patients were identified for mortality analysis. In general, the mortality rates during the first year of ART among patients with baseline CD4 counts <350 cells/μL significantly decreased through the changes of ART-eligible policies (Figure 4(A)). The cumulative probability of death among different groups of baseline CD4 counts was significantly different after initiation of ART. At the end of the first year, the probabilities of death were 5.53, 2.08, 0.59, 0.27, and 0.23% in patients with baseline CD4 counts <50, 50–199, 200–349, 350–499, and ≥500 cells/µL, respectively. With the increase of ART duration, the corresponding death probabilities persistently increased to 11.9, 8.99, 5.38, 3.98, and 1.81%, respectively, after 10 years of ART (Figure 4(B)). Note that 26 patients with baseline CD4 > 500 cells/µL that underwent ART were followed up for 10 years. The cumulative mortality was much higher in patients with baseline CD8 counts <500 cells/µL than that in patients with baseline CD8 counts of 500–1000 cells/µL (*p*<0.001) and ≥1000 cells/µL (*p*<0.001) (Figure 4(C)). In addition, at each follow-up period, the highest probabilities of death were observed in patients with baseline CD4/CD8 ratio <0.1, followed by patients with baseline CD4/CD8 ratio of 0.1–0.19. Patients with CD4/CD8 ratio of 0.2–0.39 had similar cumulative probabilities of death as patients with CD4/CD8 ratio ≥0.4 (*p* = 0.09; Figure 4(D)).

**Figure 5.**
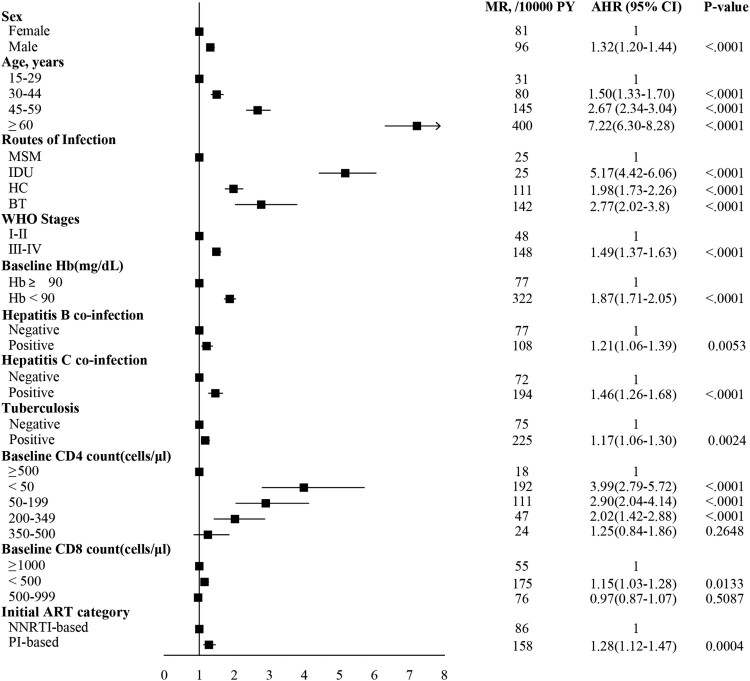
Cox proportional hazard regression analysis to identify factors associated with mortality. For regression analysis, the mortality rate per 10,000 person-years was adjusted for age, sex, infection route, WHO clinical stages, HBsAg positive, Anti-HCV positive, tuberculosis, baseline hemoglobin, baseline CD4 count, baseline CD8 count, and ART regimen. The rate difference was calculated as the adjusted mortality rate of patients on ART. The boxes represent hazard ratios, the horizontal lines represent 95% CI. MSM, men who have sex with men; HC, heterosexual contact; IDU, injecting drug users; BT, blood transfusion; Hb, hemoglobin; ART, antiretroviral therapy; MR, mortality rate; AHR, adjusted hazard ratio; HBsAg, hepatitis B surface antigen; Anti-HCV, antibody to hepatitis C virus; NNRTI, non-nucleoside reverse transcriptase inhibitor; PI, protease inhibitor.

With the multivariable Cox proportional hazard model, we found that male, older age, lower baseline hemoglobin and CD4 counts and CD8 counts, advanced WHO clinical stage, HBsAg positive, positive of antibody to hepatitis C virus, tuberculosis, and PI-based regimen were associated with a high risk of mortality (Figure 5).

### Binary-indicator immune restoration and death probabilities

**Figure 6.**
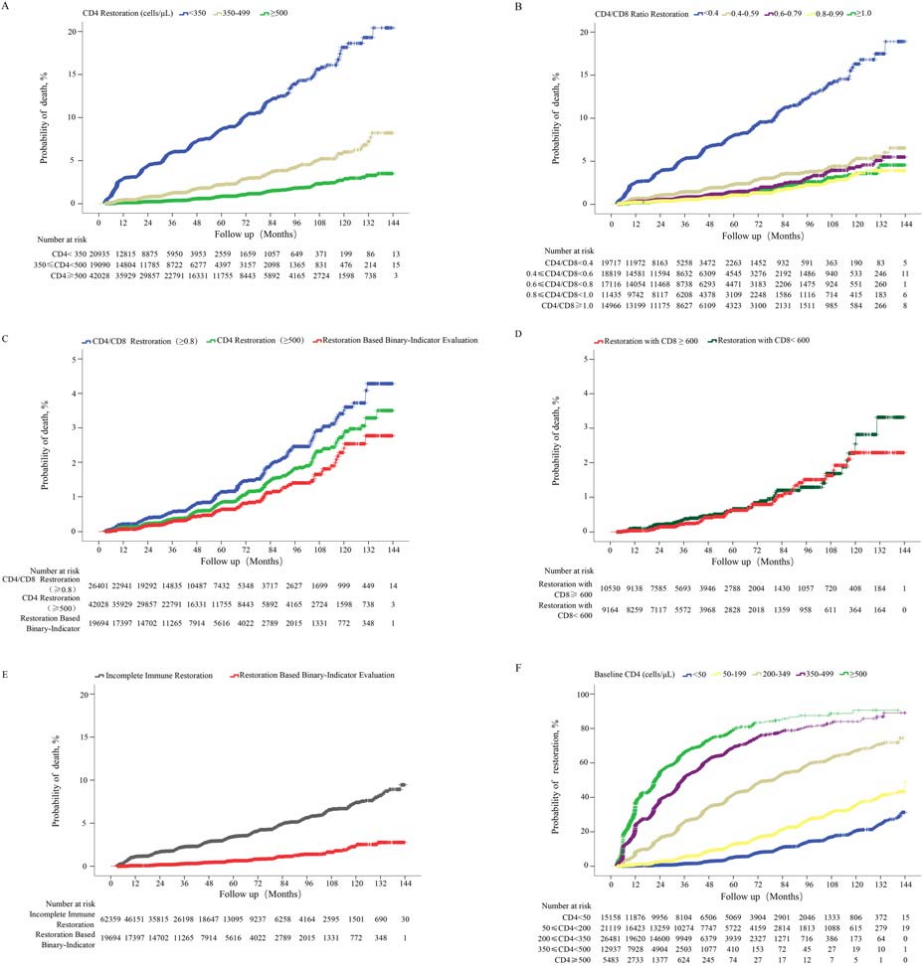
Comparison of cumulative probability of death among HIV-1-infected patients with different immune restoration status. Comparison of the cumulative probability of death between HIV-1-infected patients achieving CD4 counts ≥350, 350–499, and ≥500 cells/μL (A), CD4/CD8 ratio <0.4, 0.4–0.59, 0.6–0.79, 0.8–0.99, and ≥1.0 (B), CD4 counts ≥500 cells/μL, CD4/CD8 ratio ≥0.8, and binary indicator-based immune restoration (simultaneously achieving CD4 counts ≥500 cells/μL and CD4/CD8 ratio ≥0.8) (C), binary indicator-based immune restoration with CD8 counts ≥600 and <600 cells/μL (D), binary indicator-based immune restoration and incomplete immune restoration (E). The cumulative probability of binary indicator-based immune restoration among patients with different baseline CD4 counts (F).

A total of 1,617 deaths from the immune analysis group were selected for comparative analysis of the probabilities of death. Firstly, we found that patients achieving CD4 counts ≥500 cells/µL among different CD4 count restoration strata (*p* <0.0001 for pairwise comparison; Figure 6(A)) or CD4/CD8 ratio ≥0.8 among different CD4/CD8 ratio restoration strata (*p* = 0.501 for comparison of CD4/CD8 ratio 0.8–0.99 and ≥1.0, while *P* < 0.05 for comparison of CD4/CD8 ratio 0.8–0.99 vs. each group with CD4/CD8 ratio < 0.8; Figure 6(B)) had the lowest probability of death. Next, we found that patients achieving binary-indicator immune restoration had lower probabilities of death than patients achieving CD4 counts ≥500 cells/µL (2.77% vs 3.50%, *p* = 0.020; Figure 6(C)), or CD4/CD8 ratio ≥ 0.8 (2.77% vs 4.28%, *p* <0.001; Figure 6(C)) after 12 years of ART. The calculated threshold of CD8 count was approximately 600 cells/µL when the CD4 count was 500 cells/µL and the CD4/CD8 ratio was 0.8. We further compared the probabilities of death between patients achieving CD4 counts ≥500 cells/µL and CD4/CD8 ratio ≥0.8, with CD8 counts <600 cells/µL or CD8 counts ≥600 cells/µL, and found no significant difference between the two groups (*p*> 0.05; Figure 6(D)). Finally, we found that patients achieving binary-indicator immune restoration had lower probability of death than not achieving this binary-indicator goal (2.77% vs 8.93%, *p* <0.001; Figure 6(E)).

Based on the above comparisons of probability of death, we proposed a new assessment system for immune restoration, which was defined as the simultaneous achievement of CD4 counts ≥500 cells/µL and CD4/CD8 ratio ≥0.8. Finally, 18,819 of 81,178 patients achieved the binary-indicator based therapy goal; the cumulative probability of achieving the new therapy goal in patients with baseline CD4 counts <50, 50–199, 200–349, 350–499, and ≥500 cells/μL was 31.29, 49.00, 74.38, 89.06, and 90.60%, respectively, after 12 years of ART (Figure 6(F)), which was lower than that of achieving CD4 counts ≥500 cells/μL alone at the paralleled follow-up points. Note that 22 patients with baseline CD4 counts 350–499 cells/μL and 5 patients with baseline CD4 counts ≥500 cells/μL were followed up for 12 years, respectively. At last, except for one patient in group of baseline CD4 counts 350–499 cells/μL, all patients in both of those two groups had achieved the binary-indicator based therapy goal before 12th year of study period.

## Discussion

In this study, we comprehensively analyzed the dynamics of cumulative immune restoration, overall mortality, and associated risk factors among 91,805 patients who underwent 12 years of ART in China. Our findings showed that: (1) long-term ART resulted in different degrees of immune restoration, reflected by a continuous increase of CD4 counts and CD4/CD8 ratio, as well as partial restoration of CD8 counts, (2) through expanding ART eligibility criteria and optimizing ART regimen for PLWH, the mortality rate could be significantly reduced, and (3) compared with a single immune parameter-based system, a binary indicator-based immune assessment system (CD4 counts ≥500 cells/µL and CD4/CD8 ratio ≥0.8) could more accurately evaluate the extent of immune restoration and predict the probabilities of death in the era of “treat all.”

We found that patients with baseline CD4 counts <200 cells/μL, especially with baseline CD4 counts <50 cells/μL, were unable to reach normalized CD4 counts even after a decade of ART compared to those with CD4 counts ≥350 cells/μL. Poor CD4 counts restoration may possibly be associated with increased intrinsic CD4 loss, pathological lesions in the lymphoid tissues, and thymic and lymphoid differentiation dysfunction, particularly at the late stages of disease, even in patients with persistent viral suppression after ART [20–24]. Further, we also found that the CD8 counts remained above the normal level in HIV-1 patients receiving long-term ART, regardless of their CD4 counts. Currently, available evidence suggests that ART can only induce partial normalization of the frequency, activation, and diversity of global CD8 T cells [25]. For patients with undetectable HIV RNA, overactivation and expansion of CD8 T cells, as well as systemic inflammatory lesions could be reduced, but not eliminated due to the presence of HIV-1 antigens and gut microbiota perturbation/translocation [26]. Similar to CD4 counts, we found that the CD4/CD8 ratio showed an increasing trajectory after ART initiation; however, majority of the patients were unable to achieve a ratio of 0.8 due to the high levels of CD8 counts and insufficient CD4 counts restoration. It has been reported that there is a negative correlation between CD4/CD8 ratio and T-cell senescence and activation/exhaustion despite peripheral CD4 restoration [27]. Furthermore, several studies have also shown that incomplete recovery of CD4 counts, CD8 counts, or CD4/CD8 ratio is independently associated with a higher risk of severe outcomes, such as AIDS- or non-AIDS-related events and death [6,7,28–33]. In summary, CD4 counts, CD8 counts, and CD4/CD8 ratio reflect the immune status and predict different outcomes.

Previously, the restoration of CD4 cell counts was used as the major indicator of successful immune restoration [32]. In the era of “treat all,” some patients with HIV start their ART at CD4 counts ≥500 cells/µL. Hence, multiple immune parameters are required to assess immune restoration in such patients. In 2017, Lee and colleagues [34] proposed achieving CD4 ≥ 500 cells/μL and CD4:CD8 ratio ≥0.8 concurrently as an optimal immune outcome to investigate related factors. However, their study did not provide evidence for identifying cutoff values of CD4 count and CD4:CD8 ratio, and did not investigate the association between achieving binary-indicator immune recovery and prognosis. In this study, for the first time, we proposed the best cutoff value of CD4 counts (≥500 cells/μL) and CD4/CD8 ratio (≥0.8) in this binary-indicator assessment, based on comparison of mortality in the setting of various immune restoration. We found that the cumulative probability of binary-indicator immune restoration in patients was lower than that of CD4 ≥ 500 cells/μL alone. Furthermore, we found that patients achieving both CD4 counts ≥500 cells/µL and CD4/CD8 ratio ≥0.8 had lower probabilities of death than patients achieving CD4 counts ≥500 cells/µL, or CD4/CD8 ratio ≥0.8, or not achieving this binary indictor-based goal after 12 years of ART. Several studies confirmed that achieving CD4 counts ≥500 cells/µL was associated with a lower mortality [29,35]. Although few studies investigated the association between CD4/CD8 ratio restoration ≥0.8 and mortality, Demontès M, et al. [36] reported multimorbidity was associated with CD4/CD8 ratio < 0.8, and Marianna M, et al. [37] found CD4/CD8 ratio < 0.8 could be used as a predictor for cardiovascular diseases. This new system could have several important implications in clinical practice: (1)it could guide physicians, healthcare providers, and social workers to accurately assess the immune status upon ART, and retarget those patients who were considered to have achieved immune restoration based on normalized CD4 counts alone, (2) in the era of “treat all”, using CD4 cell counts as the only immune evaluation parameter may lead to the overestimation of successful immune restoration and neglection of the risk of death in patients with HIV, (3) the high probability of incomplete immune reconstitution after long-term treatment implies that only ART might not be sufficient to achieve complete immune restoration.

Globally, the annual number of deaths from AIDS-related illness among PLWH has fallen from a peak of 1.7 million in 2004 (initiating ART) to 770,000 in 2018, as reported by UNAIDS. Based on the NFATP database, we also observed a significant decrease in mortality rates within the first year of ART, based on each ART-eligible policy for PLWH with baseline CD4 counts <350 cells/μL, especially for PLWH with baseline CD4 counts <200 cells/μL. This observation could be attributed to the in-time change of ART-eligible policy, the increasing coverage of care, the introduction of new treatment drugs, and the optimization of treatment regimen in China.

This study has certain limitations. First, clinical data, including HIV RNA, clinical symptoms, opportunistic infections, and cause of death were not mandatory fields for the NFATP database. This may partially influence the analysis of factors associated with immune restoration and mortality. Second, due to the low quality of data, such as high proportion of missing variables, frequent loss of follow-up in some hospitals, and so on, this study enrolled patients in 17 tertiary hospitals. The study participants accounted for approximately one-tenth of all PLWH receiving ART and male participants accounted for over than 80%, which well-represented the regional and gender distribution of HIV-1 patients in China. However, there is still an inevitable selection bias in our study. Third, in this real-world study, certain unmeasured confounders and censored data may have influenced the results. Fourth, the eligibility of free ART for HIV-1 patients in China was CD4 < 500 cells/μL since 2014, and changed to all infected patients regardless of CD4 count since 2016, which can explain why only small number of patients with baseline CD4 ≥ 350 cells/µL underwent 10 years of ART treatment in our cohort.

In conclusion, the findings of our study indicate that all HIV-1 patients on long-term ART achieve different degrees of immune restoration. Here, we proposed that a new binary indicator-based immune assessment system (CD4 counts ≥500 cells/μL and CD4/CD8 ratio ≥0.8) could more accurately assess the function and status of the immune system and predict the probability of death after long-term ART in the era of “treat all.” However, further validation is needed to confirm its applicability.

## Supplementary Material

Clean_copy_of_appendix.doc
