# Supplementary material for: Immune restoration in HIV-1-infected patients after 12 years of antiretroviral therapy: a real-world observational study
Source: Emerg Microbes Infect. 2020 Dec 7;9(1):2550–61. doi: 10.1080/22221751.2020.1840928 (PMC7733958; doi:10.1080/22221751.2020.1840928)
Supplement: Clean_copy_of_appendix.doc [file TEMI_A_1840928_SM4542.doc]

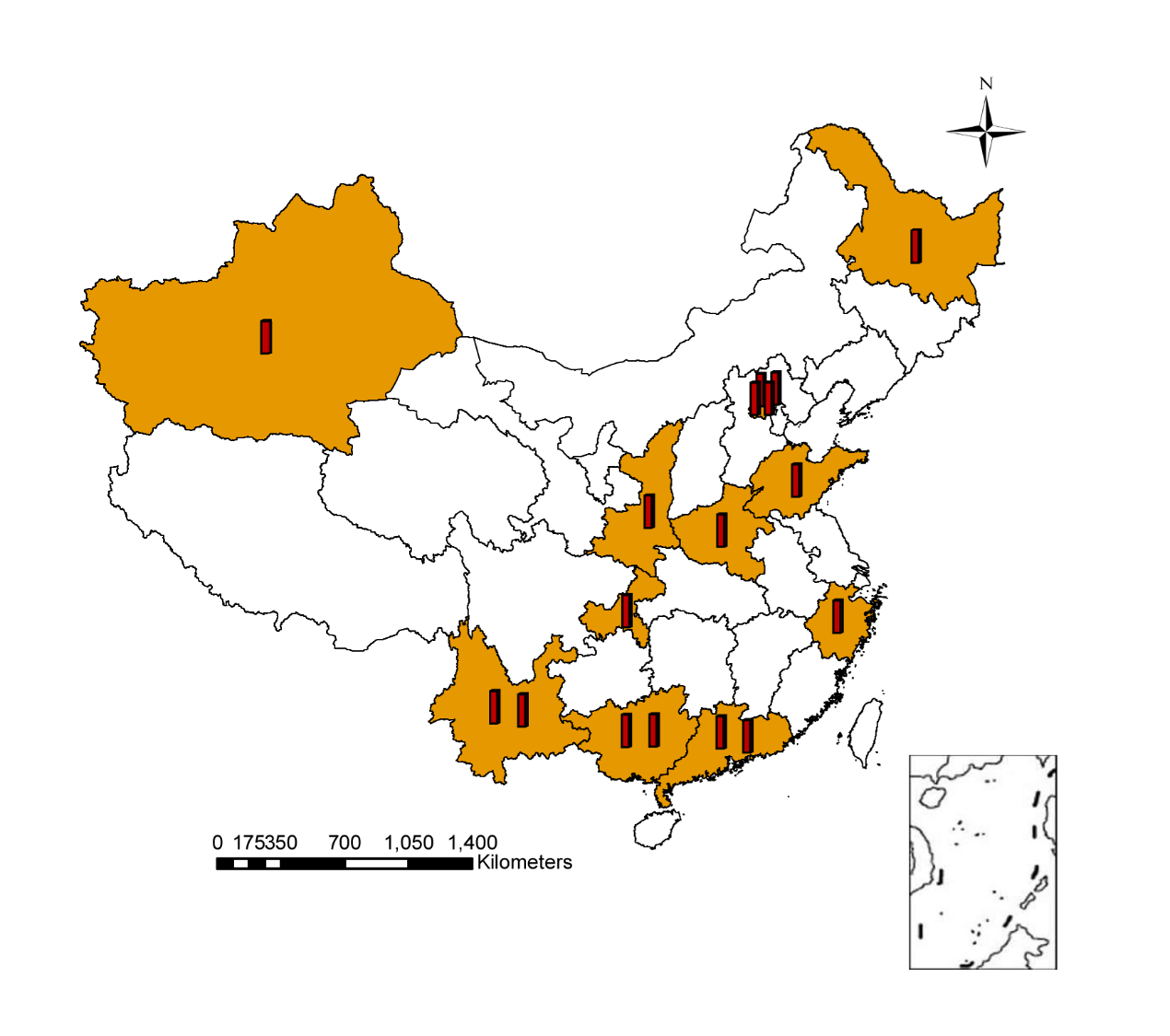


Appendix 1 The locations of 17 hospitals included in the study.

Appendix 2 Three analyses in this study

We conducted three analyses in this study. First, we assessed the dynamics of CD4 counts, CD8 counts, CD4/CD8 ratio at baseline and different time points after ART initiation stratified by the baseline CD4 counts: <50, 50–199, 200–349, 350–499, and ≥500 cells/μL. The values of CD4 counts, CD8 counts, CD4/CD8 ratio were expressed as median and interquartile range (IQR).

Second, we assessed the probability of CD4 cell counts recovery, CD4/CD8 ratio restoration, and binary-indicator immune restoration (CD4 counts ≥500 cells/µL and CD4/CD8 ratio ≥0.8), and explored the factors associated with achieving these events. We estimated these probabilities using Kaplan-Meier analysis stratified by the baseline CD4 counts, and determined statistical significance using the log-rank test for running parallel hazard rates and a two-stage procedure for crossing hazard rates. The incidence rate of restoration was calculated by dividing the number of events by the total number of person-years of follow-up. The Cox proportional hazard model was used to identify factors independently associated with restoration. Covariates were included in the multivariable model if they had a p value less than 0.1 in univariable analysis.

Third, we assessed the probability of death using Kaplan-Meier analysis and explored factors associated with death using the Cox proportional model. The stratified groups in the analysis of probability of death, the covariates in the Cox proportional model, the calculation of mortality rate, and the methods for group comparisons were similar to those in the analysis of immune restoration. In addition, we compared the probability of death between patients stratified by achieving different thresholds of CD4 counts or CD4/CD8 ratio and those stratified by achieving binary-indicator immune restoration.

Appendix 3 The corresponding times when 50% (probability) and 80% of patients with different baseline CD4 counts achieved CD4 counts ≥200, ≥350, and ≥500 cells/uL within the longest follow-up duration of 12 years

| Baseline CD4（cells/uL） | Achieving CD4 ≥200 | | Achieving CD4 ≥350 | | Achieving CD4≥500 | |
| --- | --- | --- | --- | --- | --- | --- |
|  | 50%  (Months) | 80%  (Months) | 50%  (Months) | 80%  (Months) | 50%  (Months) | 80%  (Months) |
| <50 | 17.3 | 33.1 | 50 | 90.5 | 87.4 | None |
| 50-199 | 5.2 | 14.9 | 23.5 | 55 | 61.3 | 134.3 |
| 200-349 | - | - | 5.6 | 14.2 | 18.8 | 49.9 |
| 350-499 | - | - | - | - | 6 | 18.1 |

Appendix 4 Cox proportional hazard regression analysis to identify factors associated with CD4 counts ≥500 cells/μL

| Factors | CD4≥500 (n) | Incidence, /100 PY | HR (95% CI) | P-value | AHR (95% CI) | P-value |
| --- | --- | --- | --- | --- | --- | --- |
| Total | 40587 | 24.35 |  |  |  |  |
| Sex |  |  |  |  |  |  |
| Male | 32126 | 24.37 | 0.98 (0.95-1.00) | 0.064 | 0.84(0.81-0.86) | <.0001 |
| Female | 8461 | 24.29 | 1 |  | 1 |  |
| Age at start of ART (years) |  |  |  |  |  |  |
| 15～29 | 16289 | 34.74 | 2.34 (2.22-2.47) | <.0001 | 1.73(1.64-1.83) | <.0001 |
| 30～44 | 17314 | 22.46 | 1.59 (1.51-1.68) | <.0001 | 1.6(1.52-1.69) | <.0001 |
| 45～59 | 5430 | 17.04 | 1.20 (1.14-1.27) | <.0001 | 1.32(1.25-1.4) | <.0001 |
| ≥60 | 1554 | 14.35 | 1 |  | 1 |  |
| Routes of infection |  |  |  |  |  |  |
| Injection drug use | 2149 | 15.18 | 0.81 (0.78-0.85) | <.0001 | 0.83(0.79-0.88) | <.0001 |
| MSM | 19706 | 35.65 | 1.72 (1.69-1.76) | <.0001 | 1.1(1.07-1.13) | <.0001 |
| Heterosexual | 16547 | 19.25 | 1 |  | 1 |  |
| Blood transfusion | 276 | 16.63 | 0.91 (0.81-1.02) | 0.1153 | 1.02(0.91-1.15) | 0.7467 |
| Other/Unknown | 1909 | 19.83 | 1.03 (0.98-1.08) | 0.2129 | 1.03(0.98-1.08) | 0.1894 |
| Baseline Hb (mg/dL) |  |  |  |  |  |  |
| <90 | 1414 | 10.41 | 1 |  | 1 |  |
| ≥90 | 38371 | 25.53 | 2.33 (2.21-2.45) | <.0001 | 0.95(0.9-1.00) | 0.0674 |
| Unknow | 802 | 28.95 | 2.57 (2.35-2.80) | <.0001 | 0.91(0.83-0.99) | 0.0327 |
| Baseline CD4 count (cells/μL） |  |  |  |  |  |  |
| ＜50 | 3884 | 7.51 | 1 |  | 1 |  |
| 50～199 | 7396 | 12.41 | 1.67 (1.60-1.73) | <.0001 | 1.1(1.04-1.17) | 0.0006 |
| 200～349 | 17885 | 40.25 | 5.45 (5.27-5.65) | <.0001 | 2.66(2.49-2.84) | <.0001 |
| 350～499 | 11422 | 104.5 | 14.28 (13.75-14.83) | <.0001 | 5.94(5.52-6.4) | <.0001 |
| Baseline CD8 count (cells/μL) |  |  |  |  |  |  |
| ＜500 | 6013 | 11.93 | 1 |  | 1 |  |
| 500-999 | 17810 | 24.99 | 2.01 (1.96-2.07) | <.0001 | 1.17(1.13-1.21) | <.0001 |
| ≥1000 | 16764 | 37.26 | 2.90 (2.82-2.99) | <.0001 | 1.48(1.42-1.55) | <.0001 |
| CD4/CD8 ratio at Baseline^*^ |  |  |  |  |  |  |
| < 0.10 | 4614 | 8.01 | 1 |  | - | - |
| 0.10 - 0.19 | 7679 | 18.14 | 2.20 (2.13-2.29) | <.0001 | - | - |
| 0.20-0.39 | 18226 | 37.26 | 4.40 (4.25-4.54) | <.0001 | - | - |
| ≥ 0.40 | 10068 | 56.5 | 6.54 (6.32-6.78) | <.0001 | - | - |
| HBsAg |  |  |  |  |  |  |
| Negative | 27902 | 28.27 | 1.27 (1.22-1.33) | <.0001 | 1.12(1.07-1.16) | <.0001 |
| Positive | 2629 | 21.73 | 1 |  | 1 |  |
| Unknown | 10056 | 18 | 0.89 (0.86-0.93) | <.0001 | 1.1(1.03-1.17) | 0.0046 |
| Anti-HCV |  |  |  |  |  |  |
| Negative | 28350 | 28.19 | 1.38 (1.32-1.45) | <.0001 | 1.03(0.98-1.09) | 0.2544 |
| Positive | 1802 | 19.6 | 1 |  | 1 |  |
| Unknown | 10435 | 18.35 | 0.99 (0.94-1.04) | 0.6768 | 0.97(0.9-1.05) | 0.426 |
| Tuberculosis |  |  |  |  |  |  |
| Negative | 36260 | 27.08 | 2.14 (2.04-2.25) | <.0001 | 0.97(0.92-1.02) | 0.169 |
| Positive | 1683 | 12.08 | 1 |  | 1 |  |
| Unknown | 2644 | 14.06 | 1.27 (1.20-1.35) | <.0001 | 0.96(0.9-1.02) | 0.1777 |
| Initial antiretroviral regimen |  |  |  |  |  |  |
| NNRTI-based | 37952 | 24.26 | 1 |  | 1 |  |
| PI-based | 1972 | 31.71 | 1.22 (1.17-1.28) | <.0001 | 1.43(1.37-1.5) | <.0001 |
| Other | 663 | 16.61 | 0.69 (0.64-0.75) | <.0001 | 1.08(1-1.17) | 0.044 |

ART, antiretroviral therapy; MSM, men who have sex with men; Hb, hemoglobin; HBsAg, hepatitis B surface antigen; Anti-HCV, antibody to hepatitis C virus; NNRTI, non-nucleoside reverse transcriptase inhibitor; PI, protease inhibitor. *There was collinearity between baseline CD4 counts and CD4/CD8 ratio, and so CD4/CD8 ratio was not included in the multivariable model.

Appendix 5 Cox proportional hazard regression analysis to identify factors associated with CD4/CD8 ratio ≥0.4

| Factors | CD4/CD8 ratio ≥0.4 (n) | | | Incidence, /100 PY | HR (95% CI) | p value | AHR (95% CI) | p value |
| --- | --- | --- | --- | --- | --- | --- | --- | --- |
| Total |  | | |  |  |  |  |  |
| Sex |  | | |  |  |  |  |  |
| Male | 38234 | | | 45.21 | 0.87 (0.85-0.89) | <.0001 | 0.76(0.74-0.78) | <.0001 |
| Female | 9359 | | | 52.41 | 1 |  | 1 |  |
| Age at start of ART (years) |  | | |  |  |  |  |  |
| 15～29 | 17372 | | | 61.37 | 1.45 (1.39-1.51) | <.0001 | 1.14(1.09-1.19) | <.0001 |
| 30～44 | 20192 | | | 41.84 | 1.05 (1.01-1.09) | 0.0285 | 1.02(0.98-1.07) | 0.3085 |
| 45～59 | 7508 | | | 38.07 | 0.95 (0.91-0.99) | 0.0226 | 1.00(0.95-1.05) | 0.947 |
| ≥60 | 2521 | | | 41.14 | 1 |  | 1 |  |
| Routes of infection |  | | |  |  |  |  |  |
| Injection drug use | 2821 | | | 30.9 | 0.82 (0.78-0.85) | <.0001 | 0.83(0.79-0.86) | <.0001 |
| MSM | 21548 | | | 63.41 | 1.49 (1.46-1.52) | <.0001 | 1.10(1.07-1.13) | <.0001 |
| Heterosexual | 20546 | | | 39.17 | 1 |  | 1 |  |
| Blood transfusion | 330 | | | 35.28 | 0.94 (0.84-1.05) | 0.2566 | 1.01(0.91-1.13) | 0.8011 |
| Other/Unknown | 2348 | | | 39.63 | 1.01 (0.97-1.05) | 0.6958 | 0.98(0.94-1.02) | 0.3571 |
| Baseline Hb (mg/dL) | | | |  |  |  |  |  |
| <90 | 2522 | | | 26.55 | 1 |  | 1 |  |
| ≥90 | 44130 | | | 48.32 | 1.72 (1.65-1.79) | <.0001 | 0.96(0.92-1) | 0.0373 |
| Unknow | 941 | | | 59.04 | 2.01 (1.86-2.16) | <.0001 | 1.06(0.98-1.15) | 0.1403 |
| Baseline CD4 count (cells/μL） | | | |  |  |  |  |  |
| ＜50 | 7494 | | | 18.54 | 1 |  | 1 |  |
| 50～199 | 13466 | | | 38.37 | 2.03 (1.97-2.08) | <.0001 | 2.75(2.66-2.84) | <.0001 |
| 200～349 | 17687 | | | 88.14 | 4.48 (4.36-4.60) | <.0001 | 7.88(7.59-8.19) | <.0001 |
| 350～499 | 6874 | | | 100 | 6.26 (6.06-6.48) | <.0001 | 13.41(12.81-14.04) | <.0001 |
| ≥ 500 |  | | |  |  |  | 15.43(14.54-16.37) | <.0001 |
| Baseline CD8 count (cells/μL) | | |  |  |  |  |  |  |
| ＜500 | 7999 | | | 28.49 | 1 |  | 1 |  |
| 500-999 | 18079 | | | 48.46 | 1.64 (1.59-1.68) | <.0001 | 0.71(0.69-0.73) | <.0001 |
| ≥1000 | 21515 | | | 58.09 | 1.89 (1.84-1.94) | <.0001 | 0.38(0.36-0.39) | <.0001 |
| CD4/CD8 ratio at Baseline^*^ |  | | |  |  |  |  |  |
| < 0.10 | 8039 | | | 16.78 | 1 |  | - | - |
| 0.10 - 0.19 | 11953 | | | 39.21 | 2.32 (2.26-2.39) | <.0001 | - | - |
| 0.20-0.39 | 27601 | | | 100 | 6.61 (6.44-6.78) | <.0001 | - | - |
| HBsAg |  | | |  |  |  |  |  |
| Negative | 32226 | | | 53.08 | 1.15 (1.11-1.19) | <.0001 | 1.01(0.97-1.04) | 0.8351 |
| Positive | 3317 | | | 45.34 | 1 |  | 1 |  |
| Unknown | 12050 | | | 35.03 | 0.83 (0.80-0.86) | <.0001 | 1.03(0.97-1.09) | 0.3465 |
| Anti-HCV |  | | |  |  |  |  |  |
| Negative | 32844 | | | 53.34 | 1.27 (1.21-1.32) | <.0001 | 1.08(1.03-1.14) | 0.0019 |
| Positive | 2299 | | | 40.63 | 1 |  | 1 |  |
| Unknown | 12450 | | | 35.38 | 0.92 (0.88-0.96) | 0.0002 | 0.93(0.87-0.99) | 0.0253 |
| Tuberculosis |  | | |  |  |  |  |  |
| Negative | 41649 | | | 50.91 | 1.59 (1.53-1.66) | <.0001 | 0.96(0.92-1) | 0.0547 |
| Positive | 2779 | | | 30.26 | 1 |  | 1 |  |
| Unknown | 3165 | | | 27.69 | 0.99 (0.94-1.04) | 0.5731 | 0.89(0.84-0.94) | <.0001 |
| Initial antiretroviral regimen | |  | |  |  |  |  |  |
| NNRTI-based | 44597 | | | 46.69 | 1 |  | 1 |  |
| PI-based | 2028 | | | 49.18 | 1.00 (0.96-1.05) |  | 1.08(1.03-1.13) | 0.0012 |
| Other | 968 | | | 34.86 | 0.76 (0.71-0.80) |  | 1.03(0.97-1.1) | 0.3503 |

ART, antiretroviral therapy; MSM, men who have sex with men; Hb, hemoglobin; HR, Harzard ratio; AHR, Adjusted Harzard ratio; HBsAg, hepatitis B surface antigen; Anti-HCV, antibody to hepatitis C virus; NNRTI, non-nucleoside reverse transcriptase inhibitor; PI, protease inhibitor. *There was collinearity between baseline CD4 counts and CD4/CD8 ratio, and so CD4/CD8 ratio was not included in the multivariable model.

Appendix 6 Cox proportional hazard regression analysis to identify factors associated with CD4/CD8 ratio ≥0.8

| Factors | CD4/CD8 ratio ≥0.8 (n) | | Incidence, /100 PY | HR (95% CI) | p value | AHR (95% CI) | p value |
| --- | --- | --- | --- | --- | --- | --- | --- |
| Total | 28619 | | 13.18 |  |  |  |  |
| Sex |  | |  |  |  |  |  |
| Male | 22048 | | 12.85 | 0.86 (0.83-0.88) | <.0001 | 0.79(0.76-0.81) | <.0001 |
| Female | 6571 | | 14.43 | 1 |  | 1 |  |
| Age at start of ART (years) |  | |  |  |  |  |  |
| 15～29 | 10664 | | 15.31 | 1.09 (1.04-1.1.15) | 0.0009 | 0.73(0.69-0.77) | <.0001 |
| 30～44 | 11950 | | 11.99 | 0.89 (0.84-0.93) | <.0001 | 0.79(0.75-0.83) | <.0001 |
| 45～59 | 4433 | | 12.14 | 0.89 (0.84-0.94) | <.0001 | 0.89(0.84-0.94) | <.0001 |
| ≥60 | 1572 | | 13.98 | 1 |  | 1 |  |
| Routes of infection |  | |  |  |  |  |  |
| Injection drug use | 1318 | | 7.35 | 0.66 (0.63-0.70) | <.0001 | 0.78(0.73-0.83) | <.0001 |
| MSM | 13551 | | 17.37 | 1.42 (1.38-1.45) | <.0001 | 1.10(1.07-1.14) | <.0001 |
| Heterosexual | 12181 | | 11.46 | 1 |  | 1 |  |
| Blood transfusion | 204 | | 9.47 | 0.89 (0.76-1.00) | 0.0453 | 0.93(0.81-1.07) | 0.3101 |
| Other/Unknown | 1365 | | 10.80 | 0.95 (0.90-1.01) | 0.0712 | 0.91(0.86-0.96) | 0.0005 |
| Baseline Hb (mg/dL) | | |  |  |  |  |  |
| <90 | 907 | | 5.81 | 1 |  | 1 |  |
| ≥90 | 27053 | | 13.68 | 2.24 (2.10-2.40) | <.0001 | 1.05(0.98-1.13) | 0.1935 |
| Unknow | 659 | | 18.17 | 2.91 (2.63-3.22) | <.0001 | 1.27(1.14-1.41) | <.0001 |
| Baseline CD4 count (cells/μL） | | |  |  |  |  |  |
| ＜50 | 2068 | | 3.54 | 1 |  | 1 |  |
| 50～199 | 4991 | | 7.32 | 2.10 (1.97-2.19) | <.0001 | 3.21(3.04-3.39) | <.0001 |
| 200～349 | 11579 | | 18.56 | 5.31 (5.06-5.56) | <.0001 | 12.09(11.42-12.8) | <.0001 |
| 350～499 | 6631 | | 31.81 | 9.10 (8.65-9.57) | <.0001 | 27.14(25.49-28.89) | <.0001 |
| ≥ 500 | 3350 | | 45.97 | 13.08 (12.36-13.84) | <.0001 | 49.19(45.91-52.71) | <.0001 |
| Baseline CD8 count (cells/μL) | |  |  |  |  |  |  |
| ＜500 | 4941 | | 9.58 | 1 |  | 1 |  |
| 500-999 | 14059 | | 16.03 | 1.62 (1.57-1.67) | <.0001 | 0.46(0.44-0.48) | <.0001 |
| ≥1000 | 9619 | | 12.36 | 1.22 (1.18-1.26) | <.0001 | 0.17(0.17-0.18) | <.0001 |
| CD4/CD8 ratio at Baseline^*^ |  | |  |  |  |  |  |
| < 0.10 | 2047 | | 3.08 | 1 |  | - | - |
| 0.10 - 0.19 | 3305 | | 5.89 | 2.20 (2.09-2.33) | <.0001 | - | - |
| 0.20-0.39 | 11912 | | 16.19 | 6.67 (6.36-7.00) | <.0001 | - | - |
| ≥ 0.40 | 11355 | | 54.38 | 17.28 (16.48-18.13) | <.0001 | - | - |
| HBsAg |  | |  |  |  |  |  |
| Negative | 19916 | | 15.45 | 1.18 (1.13-1.24) | <.0001 | 1.04(0.99-1.09) | 0.1219 |
| Positive | 1913 | | 12.95 | 1 |  | 1 |  |
| Unknown | 6790 | | 9.25 | 0.77 (0.73-0.81) | <.0001 | 1.08(1.00-1.17) | 0.0533 |
| Anti-HCV |  | |  |  |  |  |  |
| Negative | 20422 | | 15.65 | 1.46 (1.38-1.55) | <.0001 | 1.11(1.04-1.19) | 0.002 |
| Positive | 1177 | | 10.33 | 1 |  | 1 |  |
| Unknown | 7020 | | 9.34 | 0.95 (0.89-1.01) | 0.0998 | 0.92(0.84-1.01) | 0.0628 |
| Tuberculosis |  | |  |  |  |  |  |
| Negative | 25771 | | 14.61 | 1.96 (1.84-2.08) | <.0001 | 1.01(0.95-1.08) | 0.7247 |
| Positive | 1134 | | 7.21 | 1 |  | 1 |  |
| Unknown | 1714 | | 6.86 | 1.04 (0.96-1.12) | 0.3550 | 0.85(0.79-0.92) | <.0001 |
| Initial antiretroviral regimen |  | |  |  |  |  |  |
| NNRTI-based | 26733 | | 13.12 | 1 |  | 1 |  |
| PI-based | 1417 | | 16.71 | 1.21 (1.15-1.28) | <.0001 | 1.07(1.02-1.13) | 0.0114 |
| Other | 469 | | 9.69 | 0.75 (0.69-0.83) | <.0001 | 1.08(0.98-1.18) | 0.1201 |

ART, antiretroviral therapy; MSM, men who have sex with men; Hb, hemoglobin; PY, Person-Year; HBsAg, hepatitis B surface antigen; Anti-HCV, antibody to hepatitis C virus; NNRTI, non-nucleoside reverse transcriptase inhibitor; PI, protease inhibitor. *There was collinearity between baseline CD4 counts and CD4/CD8 ratio, and so CD4/CD8 ratio was not included in the multivariable model.
